# Pesticide poisoning in Chitwan, Nepal: a descriptive epidemiological study

**DOI:** 10.1186/s12889-017-4542-y

**Published:** 2017-07-03

**Authors:** Deepak Gyenwali, Abhinav Vaidya, Sundar Tiwari, Prakash Khatiwada, Daya Ram Lamsal, Shrikrishana Giri

**Affiliations:** 1 Farming, Health and Environment Nepal Project, Nepal Public Health Foundation, Kathmandu, Nepal; 2 Department of Community Medicine, Kathmandu Medical College, Kathmandu, Nepal; 3 Department of Entomology, Agriculture and Forestry University, Rampur, Chitwan Nepal; 4 Bharatpur Hospital, Bharatpur, Chitwan Nepal; 5 Chitwan Medical College, Teaching Hospital, Chitwan, Nepal; 6 National Academy of Medical Sciences, Bir Hospital, Kathmandu, Nepal

**Keywords:** Organophosphates, Pesticides exposure, Pesticides, Poisoning, Suicides

## Abstract

**Background:**

Globally, there is a growing concern over pesticides use, which has been linked to self-harm and suicide. However, there is paucity of research on the epidemiology of pesticides poisoning in Nepal. This study is aimed at assessing epidemiological features of pesticides poisoning among hospital-admitted cases in selected hospitals of Chitwan District of Nepal.

**Methods:**

A hospital-based quantitative study was carried out in four major hospitals of Chitwan District. Information on all pesticides poisoning cases between April 1 and December 31, 2015, was recorded by using a Pesticides Exposure Record (PER) form.

**Results:**

A total of 439 acute pesticides poisoning cases from 12 districts including Chitwan and adjoining districts attended the hospitals during the 9-month-long study period. A majority of the poisoned subjects deliberately used pesticides (89.5%) for attempted suicide. The total incidence rate was 62.67/100000 population per year. Higher annual incidence rates were found among young adults (111.66/100000 population), women (77.53/100000 population) and individuals from Dalit ethnic groups (98.22/100000 population). Pesticides responsible for poisoning were mostly insecticides (58.0%) and rodenticides (20.8%). The most used chemicals were organophosphates (37.3%) and pyrethroids (36.7%). Of the total cases, 98.6% were hospitalized, with intensive care required for 41.3%. The case fatality rate among admitted cases was 3.8%.

**Conclusions:**

This study has indicated that young adults, females and socially disadvantaged ethnic groups are at a higher risk of pesticides poisoning. Pesticides are mostly misused intentionally as an easy means for committing suicide. It is recommended that the supply of pesticides be properly regulated to prevent easy accessibility and misuse. A population-based study is warranted to reveal the actual problem of pesticides exposure and intoxication in the community.

## Background

Despite the associated benefits of pesticides use in increasing agricultural production, there have been growing concerns over the adverse effects of unsafe and inappropriate handling of pesticide to human health [1, 2]. Human exposures to pesticides are a common phenomenon in developing countries like Nepal [3–5] because of its easy access and widespread use in agriculture [6, 7]. Studies have shown that pesticides exposure has significant negative impacts on human health, this including acute severe poisoning leading to death and many chronic health issues [8, 9]. Therefore, pesticides poisoning is becoming a major public health problem worldwide [8, 10, 11].

Cases of acute pesticides poisoning — instances of suicide attempt, mass poisoning from contaminated food, chemical accidents in industries, unintended accidents and occupational exposure (agriculture) — are the most serious health hazards associated with pesticides [8, 12]. In 2002, 186,000 suicidal deaths and 4,420,000 Disability-Adjusted Life Years (DALYS) — also due to suicidal attempts using pesticides — were attributed to pesticides globally [2]. Acute pesticides poisoning is one of the major causes for emergency visits to hospitals in developing countries, including Nepal [13–18]. Deliberate self-poisoning using pesticides accounts for major proportions of all suicide cases [8, 11, 15]. Limited information is available on the pesticides poisoning situation in Nepal as it is not included in the national routine Health Management and Information System (HMIS). This study aimed to identify epidemiological characteristics of acute pesticides poisoning in Chitwan District of Nepal.

Located in the south-central part of the country, Chitwan is one among the 75 districts of Nepal. The area is known for its fertile lands, primarily used for agriculture [19]. According to the latest population and housing census, there are two municipalities and 36 Village Development Committees (VDCs) in Chitwan, which is inhabited by 579,984 people (2.2% of Nepal’s population). Chitwan has an urban-rural ratio of 1:2.05 and a male-female ratio of 1:1.08. The upper caste group (41.41%), disadvantaged ethnic groups (34.67%), relatively advantaged ethnic groups (12.02%) and Dalits (lower caste groups) (9.05%) are the major ethnic groups [20]. Agriculture is the primary occupation in the district with 30.79% of the population engaged in agro-business [21]. Chitwan ranks higher (0.551) in Human Development Index compared to other districts of Nepal and has comparatively better health, education and transportation infrastructures [22]. Chitwan and its neighboring districts are famous for commercial vegetable farming where pesticides are used extensively [23].

## Methods

We carried out a hospital-based study in four major hospitals of Chitwan. Bharatpur Hospital and Ratnanagar Hospital are public hospitals while Chitwan Medical College Teaching Hospital (CMCTH) and College of Medical Sciences Teaching Hospital (CoMSTH) are medical college hospitals. These hospitals were purposively selected as people from Chitwan and its neighboring districts mostly visit them for all medical emergencies, including poisoning conditions. Three of these hospitals are located in the district headquarter of Chitwan and provide advance life-supporting care for medical emergencies while the fourth one — Ratnanagar Hospital — is located in Ratnanagar Municipality, which is about 12 km east of the district headquarter and provides only basic care for medical emergencies.

All acute pesticides poisoning cases, as classified in accordance to World Health Organization’s (WHO) definition [24] and attending the hospitals between April 1 and December 31,2015, were selected by using a consecutive sampling method. We prospectively collected primary data by interviewing patients or their nearest kin using a modified WHO Pesticides Exposure Record (PER) form. Supplementary information was gathered by reviewing patients’ records.

In each hospital, at least two officer-level medical staffs (doctors or nurses) were designated as enumerators for data collection. The enumerators checked the patient register of the respective emergency departments every day and followed the acute pesticides poisoning cases, if any, for data collection.

Study variables included socio-demographic characteristics of poisoning cases, circumstances of poisoning, characteristics of the pesticides abused, treatment and outcome status. Descriptive analysis using Statistical Package for Social Sciences 22.0 version was used to calculate proportions and central tendencies. The Chi-square test was applied to test statistical significance of categorical variables. We reported the incidence rate of acute pesticides poisoning for Chitwan District.

## Results

From Chitwan and eleven neighboring districts, a total of 439 cases attended the hospitals due to acute pesticides poisoning in the 9 months of the study period. All cases agreed to participate in the study (response rate = 100%). Among them 308 (70.16%), 67 (15.26%), 60 (13.67%) and 4 (0.91%) cases had visited Bharatpur Hospital, CMC Teaching Hospital, CoMS Teaching Hospital and Ratnanagar Hospital, respectively. A very similar pattern in proportions (12.1–14.1%) of cases was observed in all months of study periods except during September (8.7%), November (8.7%) and December (6.4%).

### Socio-demographic characteristics of acute pesticides poisoning cases

Females constituted double the pesticides poisoning cases than the males (male: female ratio was 1:1.99) (Table 1). The mean age of the patients was 29.9 years (Standard deviation: 14.8; range: 10 months to 80 years). About three-fourth (74.3%) of the total cases were above 19 years of age with the majority being females (49.2% of the total cases). One-fifth of the cases were adolescents aged between 15 and 19 years while 5.7% were children. Two-third of the cases was females and more than two-third (71.3%) was rural residents. Disadvantaged ethnic group (37.8%), followed by upper caste group (35.5%), were found to be the major ethnic groups with higher proportions of pesticides poisoning cases.

**Table 1:** Distribution of acute pesticide poisoning cases among different groups of respondents by gender (*N* = 439)

Attributes	Male n (%)	Female n (%)	Total n (%)
Age groups			
Children (upto 14 years)	13 (2.9)	12 (2.7)	25 (5.7)
Adolescent (15–19 Years)	24 (5.5)	64 (14.6)	88 (20.0)
Adult (>19 years)	110 (25.1)	216 (49.2)	326 (74.3)
Mean Age; SD; Min- Max	29.9 Years; 14.8; 10 Months to 80 Years		
Residence			
Rural	107 (24.4)	206 (46.9)	313 (71.3)
Urban	40 (9.1)	86 (19.6)	126 (28.7)
Ethnic group			
Dalits	10 (2.3)	55 (12.5)	65 (14.8)
Disadvantaged ethnic group	59 (13.4)	107 (24.4)	166 (37.8)
Disadvantaged non-Dalit Tarai caste groups	1 (0.2)	9 (2.0)	10 (2.3)
Religious minorities	1 (0.2)	4 (0.9)	5 (1.1)
Relatively advantaged ethnic group	9 (2.1)	27 (6.2)	36 (8.2)
Upper caste group	67 (15.3)	90 (20.5)	157 (35.8)

In a majority (89.5%) of the cases, deliberate ingestion of pesticides was performed for self harm while the remaining was accidentally poisoned during its handling or during occupational activities concerning the chemicals. Among adolescent cases, 98.9% and 92.6% among adults used pesticide intentionally to commit suicide, while among children 84.0% cases were poisoned accidentally (*p* < 0.001). Other socio-demographic variables did not vary significantly from intentional and non-intentional poisoning by pesticides (Table 2).

**Table 2:** Intentional and non-intentional pesticide poisoning among different groups of respondents (*N* = 439)

Variables	Intentional (suicidal attempts) n (%)	Non-Intentional (Accidental/occupational) n (%)	*p*-value
Gender			
Male	128 (87.1)	19 (12.9)	0.235
Female	265 (90.8)	27 (9.2)	
Age groups			
Children (upto 14 years)	4 (16.0)	21 (84.0)	0.000^*^
Adolescents (15–19 Years)	87 (98.9)	1 (1.1)	
Adults (>19 years)	302 (92.6)	24 (7.4)	
Residence			
Rural	277 (88.5)	36 (11.5)	0.270
Urban	116 (92.1)	10 (7.9)	
Ethnicity			
Dalits (Lower caste group)	59 (90.8)	6 (9.2)	0.287
Disadvantaged ethnic group	152 (91.6)	14 (8.4)	
Disadvantaged non-Dalit Tarai caste group	10 (100.0)	0 (0.0)	
Religious minorities	4 (80.0)	1 (20.0)	
Relatively advantaged ethnic group	29 (80.6)	7 (19.4)	
Upper caste group	139 (88.5)	18 (11.5)	
Total	393 (89.5)	46 (10.5)	

^*^Statistically significant at *p* ≤ 0.05

### Incidence of acute pesticides poisoning (for cases from Chitwan District only)

Of the total cases during the study period of 9 months, 269 cases were from Chitwan District. The mid-year population of Chitwan District (population at risk) in the year 2015 was estimated to be 629,799 [21]. The incidence rate of acute pesticides poisoning in Chitwan District was 47.0 per 100,000 population during the 9 months of study.This is equivalent to 62.67/100,000 population per year. The annual incidence rate was higher among females (77.53/100,000 population) than among males (46.64/100,000 population). The highest recorded incidence rate was of the age group 15–29 years (111.66/100,000 population per year) followed by the age group of 30–49 years (74.39/100,000 population per year). The annual incidence was higher among urban residents (71.01/100,000 population) than among rural residents (58.59/100,000 population). Among the ethnic groups, it ranged from 50.46/100,000 in non-Dalit, Terai caste groups to 98.22/100000 population among Dalits. The incidence of intentional pesticides poisoning (55.68/100,000 population) was considerably higher than that of non-intentional pesticides poisoning (6.99/100000 population) (Table 3).

**Table 3:** Incidence Rate (per 100,000 populations per year) of acute pesticide poisoning in Chitwan District (*N* = 296)

Variables	Gender						Total		
	Male			Female					
	Cases	Population	Incidence Rate	Cases	Population	Incidence Rate	Cases	Population	Incidence Rate
Age Groups									
< 15 Years	9	95,959	12.51	6	89,737	8.91	15	185,696	10.77
15–29 Years	44	88,211	66.51	117	104,045	149.94	161	192,256	111.66
30–49 Years	29	69,025	56.02	56	83,317	89.62	85	152,342	74.39
50+ Years	24	49,863	64.18	11	49,643	29.54	35	99,506	46.90
Residence									
Urban	34	101,366	44.72	76	105,174	96.35	110	206,540	71.01
Rural	72	201,692	47.60	114	221,567	68.60	186	423,259	58.59
Ethnic Groups									
Dalits (lower castes group)	6	27,341	29.26	36	27,325	175.66	42	57,012	98.22
Disadvantaged ethnic group	44	105,123	55.81	72	104,280	92.06	116	218,359	70.83
Disadvantaged non-Dalit Tarai caste groups	1	6829	19.52	3	3445	116.11	4	10,570	50.46
Religious minorities	1	4437	30.05	3	2729	146.57	4	7400	72.07
Relatively advantaged ethnic group	8	35,739	29.85	20	36,782	72.50	28	75,680	49.33
Upper caste groups	46	123,589	49.63	56	126,336	59.10	102	260,777	52.15
Mode of Poisoning									
Intentional (Suicidal attempts)	92	303,058	40.48	171	326,741	69.78	263	629,799	55.68
Non-Intentional Accidents/occupational	14	303,058	6.16	19	326,741	7.75	33	629,799	6.99
Total Incidence among male							106	303,058	46.64
Total Incidence among female							190	326,741	77.53
Total Incidence							296	629,799	62.67

### Circumstances of pesticides poisoning

The majority (43.7%) of the cases were poisoned during evening hours (18.01 to 24.00 PM) followed by day time (30.4%) and morning hours (24.1%). The median time of poisoning was 17.22 PM while the mode was 22.00 PM at which 8% of the total cases were poisoned from pesticides. Nearly all cases (99.1%) got poisoned in their own homes. Most (98.2%) of the cases got exposed to pesticides through oral route (Table 4).

**Table 4:** Circumstances of pesticide poisonings and types of pesticides

Descriptions	Frequency	Percent
Timing of pesticide exposure (*n* = 316)		
Morning (6.00–12.00)	76	24.1
Day time (12.01–18.00)	96	30.4
Evening (18.01–24.00)	138	43.7
After mid night (0.01–5.59)	6	1.9
Median hour: 17:22, Mode: 22.00 h		
Place of pesticide exposure (*n* = 439)		
Home	435	99.1
Farm/Field	4	.9
Route of exposure (Multiple response, *n* = 439)		
Oral	431	98.2
Dermal	10	2.3
Respiratory	3	0.7
Types of pesticides (*n* = 439)		
Insecticide	263	59.9
Rodenticide	91	20.8
Herbicide	15	3.4
Fungicide	5	1.1
Unknown	65	14.8
Chemical types (multiple response in some cases due to mixed composition of chemicals)		
Organophosphorus	174	39.6
Pyrethroid	154	35.1
Phosphide (Zinc and Auminium)	95	21.6
Unknown chemical	73	16.6
Dinitrophenol derivative	4	1.0
Carbamate/thiocarbamate	2	0.5
Oraganochlorine	2	0.5

The pesticides responsible for poisoning were mostly insecticides (59.9%) and rodenticides (20.8%). Herbicides and fungicides comprised only a small proportion of the cases. The common constituent chemicals were: organophosphate compounds (39.6%), pyrethroids (35.1%) and phosphides (21.6%). Carbamates, organochlorides and dinitrophenyl derivatives contained in smaller proportions (Table 4).

### Management of acute pesticides poisoning cases and their outcomes

After exposure to pesticides, the poisoned cases arrived at the hospital within an average of 2 h (median time). Time elapsed between exposure and arrival at the hospital ranged from 10 min to 22 h. While 63 (14.4%) of the cases arrived within 1 h, more than half (59.9%) of the patients reached between 1 and 3 h and a fourth of them consulted a doctor after 3 h or more. While most of the cases (97.0%) were hospitalized in the same hospital where they were first taken to, 10 (2.3%) were immediately referred to other hospitals (hospitals other than the ones mentioned in the study) and 3 (0.7%) were discharged after few hours of observation without being admitted. The median hospital stay duration among total hospitalized cases was 4.0 days, (range: 1–24 days).

Among the hospitalized cases, 176 (41.3%) were treated in an Intensive Care Unit (ICU). The median duration of ICU stay was 4.0 days (range: 1–17 days). Among these cases, 4.1% had local effects, 93.4% had shown systemic effects and 1.4% had both local and systemic effects of pesticides poisoning. In terms of severity, 29.6% were in severe condition, while 32.6% had moderate symptoms and 32.8% had minor symptoms.

Among the total hospitalized cases due to pesticides poisoning, 372 (87.3%) cases recovered while 16 (3.8%) died. The outcome status of 38 (8.9%) cases remained unknown because they either could not be tracked after referral or they left against medical advice (LAMA) or absconded from hospitals. The case fatality rate, therefore, is 4.1%, after excluding the cases whose outcome statuses were not known (Table 5).

**Table 5:** Management of cases and outcome

Description	Frequency	Percent
Time elapsed since exposure to hospital^a^		
< 1 h	63	14.4
1-3 h	263	59.9
> 3 h	113	25.7
Hospitalization		
Yes	426	97.0
No	3	0.7
Referred to other hospitals	10	2.3
Treatment in ICU^b^		
Yes	176	41.3
No	250	58.7
Total day of ICU stay^c^		
≤ 3 days	79	44.9
3-7 days	64	36.4
> 7 days	33	18.8
Effects^a^		
Local	18	4.1
Systemic	410	93.4
Both	6	1.4
Severity^a^		
None	17	3.9
Minor	144	32.8
Moderate	143	32.6
Severe	130	29.6
Total days of hospital stay^b^		
≤ 3 days	228	53.5
3-7 Days	84	19.7
> 7 days	114	26.8
Outcome^b^		
Recovered	372	87.3
Death related	16	3.8
Unknown	38	8.9

^a^Denominator: Total cases (*n* = 439)

^b^Denominator: Hospitalized cases (*n* = 426)

^c^Denominator: ICU cases (*n* = 176)

## Discussion

Acute poisoning by using pesticides remains to be a major cause for emergency visits to the hospitals in developing countries [8, 13, 16, 18, 25, 26]. This prospective hospital-based study explored the situation of pesticides poisoning in an agriculture intensive-district of Nepal. The study population was the total number of pesticides poisoning cases who had visited the hospitals during the study period.

This study showed female predominance in acute pesticides poisonings, which was also a result revealed through prior studies conducted in Nepali hospitals [14, 18, 27]. Not just in the case of pesticides poisoning, females have outnumbered males in acute poisonings of other kinds as well, studies have found [28, 29]. Women are more vulnerable to suicide attempts than their male counterparts in developing countries due to factors like domestic violence, abusive spouse, problematic love and marital relationships and unfavorable socio-cultural practices [7, 25, 30]. Moreover, females are more likely to be engaged in impulsive acts of self-harm [7]. In contrast to higher rural suicidal rates found in several Asian countries [8, 31], a lower rate of pesticides poisoning was observed among rural residents in Chitwan. Higher rates of pesticides poisoning might have been observed among urban residents due to classification bias in defining rural and urban settings as the municipalities of Chitwan District also include villages in the process of urbanizing. The rates were higher among socially disadvantaged ethnic groups like the Dalits (lower caste groups). Social disadvantages, economical deprivation and inequalities are positively associated with higher rates of suicide [8, 32, 33]. As in other studies conducted in Nepal, the study also found young adults (15–29 years) to be highly affected by acute pesticides poisonings [14, 17, 18, 25, 27]. This age group of the population has been found to be at a high risk of self-poisoning from pesticides for attempted suicide in previously conducted South-Asia-based studies as well [7, 25]. Moreover, this age group includes adolescents, students and early career beginners who usually face psycho-emotional problems involving failure in academics, unemployment, economic hardship, difficult love affairs, family pressure etc. Such issues give individuals a negative outlook towards life and are positively associated with suicide attempts [25, 26, 30].

Due to lack of defined population at risk, the calculation of incidence rate for acute pesticides poisoning is difficult in hospital-based studies. Almost all cases of medical emergencies including pesticides poisoning from Chitwan District generally attend or are referred from other hospitals to the study hospitals. The calculated incidence rate of acute pesticides poisoning in Chitwan District comprised only of cases who attended the study hospitals. So, this study might have missed cases who did not consult the study hospitals or those who stayed at their homes ignoring minor symptoms of acute pesticides poisoning which is mostly common in occupational pesticides exposure [34, 35] or those who died before reaching the hospitals. So, the actual incidence rate of acute pesticides poisoning in Chitwan District could be higher than that was calculated in this study.

The study revealed that pesticides poisonings were more likely to occur during evening hours. Almost all cases had been exposed to pesticides in their own homes and most of them intentionally self poisoned to attempt suicide. Pesticides are becoming the first choice of method of suicide in many developing countries [11, 18, 27, 29]. The possible explanation of the timing of pesticides consumption could be psychological conditions such as stress and loneliness during evening hours. Easy access to pesticides at home may stimulate suicidal ideation [36–38]. Even in agriculture-based developing countries, the proportion of acute pesticides poisoning from occupational exposure is very low when compared to the use of pesticides for intended self harm [14, 39]. In Nepal and other under-developed countries, farmers in rural regions are engaged in subsistence level agriculture. Although there is pesticides act and regulation to regulate and control of pesticides handling and trade in Nepal [21], in practice, there is no restriction on buying pesticides from the market; and people mostly store their own supply of pesticides within the premises of or nearby their homes. Therefore, easy availability and accessibility of pesticides increases intentional use of pesticides for suicide attempts [5, 7, 8, 25, 36]. One study in rural China indicated that the availability of pesticides at home can trigger suicidal attempts among those who really did not want to die [38].

Farmers can have minor acute symptoms due to occupational pesticides exposure as expected symptoms, and hence, they may not generally seek medical consultation [34, 35]. In contrast to this, in case of intentional use of pesticides and accidental exposure, family members seek emergency medical services immediately; even if the swallowed dose of pesticide is not fatal. This could be a reason why the study has shown a very small proportion of occupational pesticides poisoning. The most commonly used pesticides for poisoning were insecticides and rodenticides; common chemical types were organophosphorous, pyrethroids and zinc phosphides. Other studies in Nepal also concluded with similar results [14, 18, 27]. These chemicals are the mostly used pesticides in Nepal. Zinc phosphide, a rodenticide is readily available even in general convenience stores in Nepal.

Chitwan District has easy access to medical care compared to many other parts of Nepal. So, most of the cases visited hospital within a short period of exposure to pesticides and before complications arose. The fatality rate was lower in comparison to the findings from previous studies in Nepal and other Asian countries [12, 14, 15, 18]. Timely consultation with doctors and the availability of appropriate care in hospitals may have contributed to lowering the rate of fatality from pesticides poisoning.

This hospital-based study might have missed out on cases who did not consult the study hospitals. Similarly, data from one complete year could not be included in the report to show seasonal and monthly variations of pesticides poisoning. So, the estimated annual incidence rate could have been different if the actual pattern of cases was different in the remaining 3 months than in the study period. This study was only focused on poisoning due to pesticides. So, the result could not be compared with other kinds of poisoning and with suicidal attempts by other methods. These may be considered as limitations of this study.

## Conclusions

Pesticides are intentionally misused as an easy means for committing suicide. The findings indicated that young adults, females and socially disadvantaged ethnic groups like lower caste groups (Dalits) are at a higher risk of pesticide poisoning. Self poisoning by using pesticides usually occurs at home and during evening hours. The commonly available insecticides (organophosphates and pyrethroids) and rodenticides (zinc phosphides) are the common pesticides that cause poisonings. As this study was limited to hospital settings, a population-based study is needed to reveal the actual problem of pesticide exposure and intoxication. Strong regulatory mechanism with effective implementation of existing pesticide regulation is needed for safe handling/storage of pesticides and to limit easy access of pesticides to the general public. As the pesticides are misused for committing suicides, there is a need for preventive and mental health programs for the vulnerable groups.

## Abbreviations

CISU: Civil Society in Development
CMCTH: Chitwan Medical College Teaching Hospital
CoMSTH: College of Medical Sciences Teaching Hospital
DALYS: Disability-Adjusted Life Years
NHRC: Nepal Health Research Council
PER: Pesticide Exposure Record
SPSS: Statistical Package for Social Scientists
VDC: Village Development Committee
WHO: World Health Organization
WHO, SEARO: World Health Organization, South East Asian Regional Office
